# Small RNA signatures of the anterior cruciate ligament from patients with knee joint osteoarthritis

**DOI:** 10.3389/fmolb.2023.1266088

**Published:** 2023-12-21

**Authors:** Yalda A. Kharaz, Danae E. Zamboulis, Yongxiang Fang, Tim J. M. Welting, Mandy J. Peffers, Eithne J. Comerford

**Affiliations:** ^1^ Department of Musculoskeletal Ageing Sciences, Institute of Life Course and Medical Sciences, University of Liverpool, Liverpool, United Kingdom; ^2^ Comparative Biomedical Sciences, Royal Veterinary College, London, United Kingdom; ^3^ Centre for Genomic Research, Institute of Integrative Biology, University of Liverpool, Liverpool, United Kingdom; ^4^ Department of Orthopaedic Surgery, Maastricht University Medical Centre, Maastricht, Netherlands; ^5^ Institute of Veterinary and Ecological Sciences, University of Liverpool, Liverpool, United Kingdom

**Keywords:** anterior cruciate ligament, osteoarthritis, microRNA, small nuclear RNA, small nucleolar RNA

## Abstract

**Introduction:** The anterior cruciate ligament (ACL) is susceptible to degeneration, resulting in joint pain, reduced mobility, and osteoarthritis development. There is currently a paucity of knowledge on how anterior cruciate ligament degeneration and disease leads to osteoarthritis. Small non-coding RNAs (sncRNAs), such as microRNAs and small nucleolar RNA (snoRNA), have diverse roles, including regulation of gene expression.

**Methods:** We profiled the sncRNAs of diseased osteoarthritic ACLs to provide novel insights into osteoarthritis development. Small RNA sequencing from the ACLs of non- or end-stage human osteoarthritic knee joints was performed. Significantly differentially expressed sncRNAs were defined, and bioinformatics analysis was undertaken.

**Results and Discussion:** A total of 184 sncRNAs were differentially expressed: 68 small nucleolar RNAs, 26 small nuclear RNAs (snRNAs), and 90 microRNAs. We identified both novel and recognized (miR-206, -365, and -29b and -29c) osteoarthritis-related microRNAs and other sncRNAs (including SNORD72, SNORD113, and SNORD114). Significant pathway enrichment of differentially expressed miRNAs includes differentiation of the muscle, inflammation, proliferation of chondrocytes, and fibrosis. Putative mRNAs of the microRNA target genes were associated with the canonical pathways “hepatic fibrosis signaling” and “osteoarthritis.” The establishing sncRNA signatures of ACL disease during osteoarthritis could serve as novel biomarkers and potential therapeutic targets in ACL degeneration and osteoarthritis development.

## 1 Introduction

Ligaments are resilient connective tissues essential for bone-to-bone connections within joints (Rumian et al., 2007). The anterior cruciate ligament (ACL) is the most commonly damaged ligament (Woo et al., 2000), with an incidence of approximately 68.6 ACL ruptures per 100,000 people (Gianotti et al., 2009), resulting in considerable social and economic costs (Cumps et al., 2008; Robling et al., 2009). In the USA alone, there are approximately 100,000–175,000 ACL surgeries per year, with the cost exceeding 2 billion dollars (Griffin et al., 2000; Ali and Rouhi, 2010). ACL injuries can also lead to significant functional impairment in athletes, muscle atrophy, weakness, joint instability, and meniscal lesions and are associated with the development of osteoarthritis (OA) (Kiapour and Murray, 2014; Wurtzel et al., 2017). Greater than 50% of ACL injury patients eventually develop OA, with the degree and progression of the disease being accelerated in these cases (Barenius et al., 2014; Driban et al., 2014). Moreover, reports demonstrate that there is an association between ACL degeneration and subsequent knee OA, suggesting the importance of ACL degradation in OA pathogenesis (Hasegawa et al., 2012).

There is currently substantial interest in the area of epigenetic regulation in aging, disease, and repair mechanisms in musculoskeletal tissues such as muscle (Goljanek-Whysall et al., 2012; Soriano-Arroquia et al., 2016), cartilage (Peffers et al., 2020), tendon (Millar et al., 2015; Watts et al., 2017), and ligament (Xu et al., 2016; Li et al., 2017a). Small non-coding RNAs (sncRNAs) are a class of epigenetic molecules which include microRNAs (miRNAs or miRs), small nucleolar RNAs (snoRNAs), and small nuclear RNAs (snRNAs). Small non-coding RNAs are important regulators of gene expression and are in encoded in DNA and RNA, but are not translated into proteins (Ali et al., 2021a). Their aberrant expression profiles in musculoskeletal conditions such as ACL injury are expected to be associated with cellular dysfunction and disease development (Choudhuri, 2010). We have previously identified changes in the sncRNA profiles in aging and OA human (Peffers et al., 2020) and equine cartilage (Peffers et al., 2013; Balaskas et al., 2017), aging and OA human and equine tendon (Peffers et al., 2015; Pease et al., 2017), and aging and OA murine joints and serum (Steinbusch et al., 2017). However, the pathogenesis and contribution of ACL degeneration to the development and acceleration of OA is currently unknown. Identifying sncRNAs associated with ACL degeneration and comprehending their role in OA could have an important impact on the understanding of OA pathogenesis and future management.

To date, little is known about the sncRNA changes in human diseased ACL. We hypothesize that sncRNA expression is altered in ACLs derived from OA joints in comparison to healthy joints and that their identification may elucidate the underlying mechanisms of ACL degeneration. In this study, we, therefore, undertook a non-biased approach: small RNA sequencing of ACLs from human OA knee joints and compared the findings to those of our control samples derived from human non-OA knee joints.

## 2 Materials and methods

### 2.1 Sample collection

ACLs from non-OA, healthy knee joints (control) *n* = 4 (age (mean ± standard deviation); 47.3 ± 1.7 years) were obtained from a commercial biobank (Articular Engineering) (https://www.articular.com/). Diseased OA ACLs were obtained from the knee joints of patients undergoing total knee arthroplasty for end-stage OA treatment *n* = 4 (age (mean ± standard deviation); 74.8 ± 5.4 years). Control samples were scored according to the International Cartilage Repair Society (ICRS) grading system (Van den Borne et al., 2007). RNA samples were collected and later stored at −80°C until use. In addition to the above samples, RNA from non-OA ACLS *n* = 4 (age (mean ± standard deviation); 51.2 ± 3.3 years) and diseased OA ACLs *n* = 4 (age (mean ± standard deviation); 77 ± 2.1 years) was used for validation.

### 2.2 RNA extraction

RNA extracted from ACL tissues was pulverized into a powder using a dismembranator (Mikro-S, Sartorius, Melsungen, Germany) under liquid nitrogen. Total RNA was extracted using the miRNeasy kit (Qiagen, Manchester, United Kingdom) according to the manufacturer’s instructions (Peffers et al., 2020). The RNA samples were quantified using a NanoDrop spectrophotometer (NanoDrop Technologies, Wilmington, United States). The integrity of the RNA was assessed on the Agilent 2100 Bioanalyzer (Agilent, Stockport, United Kingdom) using an RNA Pico chip (Agilent, Stockport, United Kingdom). Then, 1000 ng RNA per ACL sample was submitted for library preparation using NEBNext^®^ Small RNA Library Prep Set for Illumina (New England Biosciences (NEB), Ipswich, United States) but with the addition of a Cap-Clip™ Acid Pyrophosphatase (Cell script, Madison, United States) step to remove any 5′ cap structures on some snoRNAs (Steinbusch et al., 2017) and size selected using a range 120–300 bp (including adapters). These steps enabled both miRNAs and snoRNAs to be identified using an unbiased approach. The pooled libraries were sequenced on an Illumina HiSeq 4000 platform with sequencing chemistry version 1 to generate 2 × 150 bp paired-end reads.

### 2.3 Data processing

Read files in FASTQ format were generated from sequence data measured from one lane of an Illumina HiSeq 4000 through basecalling and de-multiplexing of indexed reads using CASAVA version 1.8.2 and adapter and quality trimming using Cutadapt version 1.2.1 (Martin, 2011) and Sickle version 1.200. Small RNA expression values were obtained through aligning reads to Ensembl GRCH38.96 human genome reference sequences using TopHat (Kim et al., 2013) version 2.0.10 with the option “–g 1”, counting aligned reads against gene features using HTSeq-count version 0.6.1p1, and filtering genes by biotype and keeping the small RNA genes for further analysis.

For mature miRNA expression profiling, the human mature miRNA sequences from miRBase (Patro et al., 2017) were used as the reference. A Salmon (Kozomara et al., 2019) index was created from these sequences, and reads shorter than 28 nucleotides in length were aligned and quantified against this index using Salmon. The obtained pseudocounts were processed and used for differential expression analysis using the DESeq2 package performed in the R environment (Love et al., 2014).

The DESeq2 analysis initially involved estimating normalization factors to account for variations in library size among the samples. Libraries were normalized using the median-of-ratios method implemented in DESeq2 (estimateSizeFactors function). Data variation across samples was modeled using negative binomial distributions. Subsequently, a generalized linear model was fitted to the data to capture the relationships between observed counts and OA status. From this model, log2 fold change values and p-values for the contrast were derived. The Benjamini–Hochberg (BH) method was used to adjust p-values and control the false discovery rate.

Counts per million (CPM) values were calculated by dividing each gene count by the total counts for that sample and subsequently multiplying by a million. Finally, small RNAs were deemed significantly differentially expressed if they met the criterion of an FDR-adjusted *p*-value of less than 5% (Benjamini and Hochberg, 1995) and a CPM of at least 10 to categorize a gene as significant.

Sequence data have been submitted to the National Centre for Biotechnology Information Gene Expression Omnibus (NCBI GEO); E-MTAB-5715. E-MTAB-9106.

### 2.4 Pathway analysis of differentially expressed miRNAs and their predicted targets

Potential biological associations of the DE miRNAs in OA ACLs were identified using Ingenuity Pathway Analysis (IPA), (IPA 2020, Qiagen Redwood City, United States) “Core Analysis.” Additionally, in order to identify putative miRNA targets, bioinformatics analysis was performed by uploading DE miRNA data into the MicroRNA Target Filter module within IPA. This analysis identifies experimentally validated miRNA–mRNA interactions from TarBase, miRecords, and the peer-reviewed biomedical literature, as well as predicted miRNA–mRNA interactions from TargetScan and creating biological networks describing functional associations. We applied a conservative filter at this point, using only experimentally validated and highly conserved predicted mRNA targets for each miRNA. Targets were then also filtered on the fibroblast cell type. Core analysis was then performed in IPA on the filtered mRNA target genes and their associated miRNAs. For each core analysis, canonical pathways, novel networks, diseases and functions, and common upstream regulators were queried. IPA z-score (statistical measure of the match between expected relationship direction and observed gene expression) is computed to infer the activation state of a predicted biological function.

Additionally, TOppGene (Chen et al., 2009) was used for overrepresentation analysis of the mRNA targets from Target Filter using Fisher’s exact test with FDR correction. This tests whether the input mRNAs associate significantly with specific pathways and generates a list of biological process gene ontology (GO) terms. Terms with FDR adjusted *p* < 0.05 were summarized using REViGO (Supek et al., 2011) with an allowed similarity of 0.4 and visualized using Cytoscape (Shannon et al., 2003).

### 2.5 qRT-PCR validation

Validation of a selected subset of small RNA sequencing results was undertaken in an independent cohort of human ACLs using real-time quantitative PCR (qRT-PCR). Total RNA was extracted as above. Small non-coding RNAs were chosen based on our current work, level of differential expression (*p* < 0.05 and logFC> 1.2), and following a literature review of differentially expressed genes (Li et al., 2017a; Kharaz et al., 2018). PolyA cDNA was synthesized using 200 ng RNA and the miScript II RT Kit. A mastermix was prepared using the miScript SYBR Green PCR Kit (Qiagen, Crawley, United Kingdom) and the appropriate bespoke designed miScript Primer Assays (Qiagen, Crawley, United Kingdom) using 1 ng/μL cDNA according to the manufacturer’s guidelines. Real-time PCR was undertaken using a LightCycler^®^ 96 system (Roche). A panel of four genes—miR-99a, miR-30c, miR-222, and SNORA46—was selected as potential reference genes because their expression was unaltered in this study. The stability of this panel of genes was assessed by applying a gene stability tool, RefFinder (Xie et al., 2012). MiR-222 was identified by NormFinder as the most stable, and the relative expression levels were normalized to miR-222 and calculated using the 2^-DCT method (Livak and Schmittgen, 2001).

### 2.6 Statistical analysis

The heatmap, volcano, and principle component analysis (PCA) plots were devised using MetaboAnalyst 3.5 (http://www.metaboanalyst.ca) which uses the R package of statistical computing software 30 (Xia et al., 2009).

## 3 Results

### 3.1 Sample assessment

The ages of the control group (non-OA, healthy knee joints) [age, mean ± standard deviation (48 ± 2.16)] and ACLs derived from OA joints (74.7 ± 5.42) were significantly different (Mann–Whitney test, *p* < 0.05), but age was not a determining factor to alter the outcome of this study (Supplementary Figure S1). Control samples had no clinical history of joint disease or osteoarthritis and were designated as control samples as they had an average International Cartilage Repair Society (ICRS) system score of 0 (Van den Borne et al., 2007).

The summary of all donors’ information is provided in Supplementary Table S1.

### 3.2 Small RNA sequencing data

Quality metrics including library depth and distribution of other small RNAs have been provided in Supplementary Table S2; Supplementary Figures S2, S3. We identified a total of 590 miRNAs, 226 snoRNAs, and 100 snRNAs (with greater than 10 CPMs in each sample).

**FIGURE 1:**
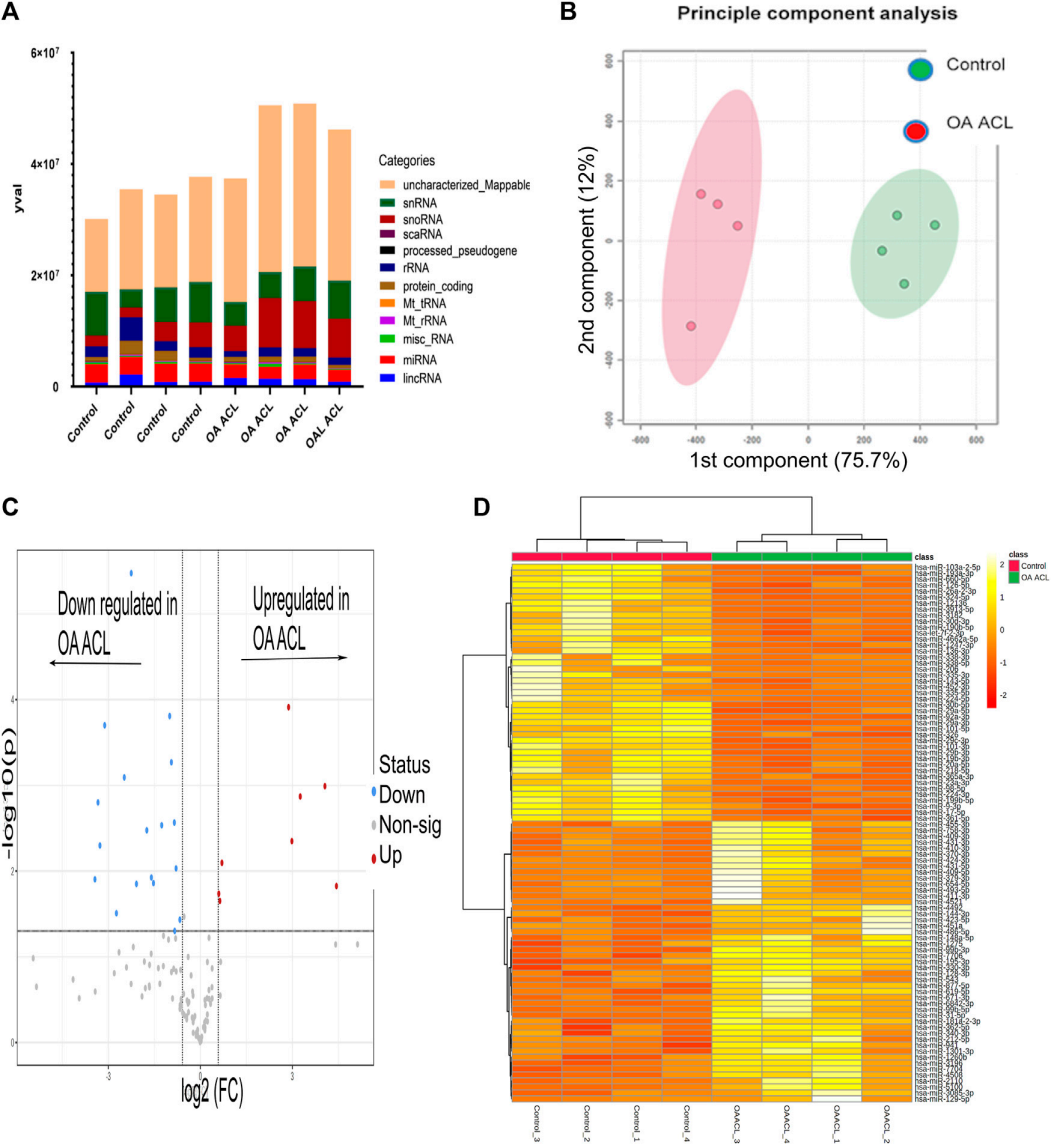
Overview of HiSeq transcriptomics data between the control and diseased OA anterior cruciate ligament. **(A)** Categories of RNAs identified in control and diseased OA anterior cruciate ligaments **(B)** Principle component analysis revealed that sncRNAs between control and diseased anterior cruciate ligaments were distinctly grouped. **(C)** Volcano plot demonstrates significant (FDR< 0.05) differentially expressed sncRNAs (red dots) with a log2 fold change>1.3. **(D)** Heatmap representation of the sncRNA reads from control and OA anterior cruciate ligaments. Columns refer to the control and OA anterior cruciate ligament samples and rows of miRNAs identified with their Ensembl identification. The color of each entry is determined by the number of reads, ranging from yellow (positive values) to red (negative values).

There were 184 differentially expressed sncRNAs identified (false discovery rate (FDR<0.05)) and with at least 10 CPMs in each sample. The categories of RNAs identified are shown in Figure 1A which included miRNAs, snoRNAs, and snRNAs. Principle component analysis (PCA) revealed that the ACLs derived from non-OA joints (control) clustered together and could be clearly separated from the ACLs derived from OA knee joints (Figure 1B).

Of the 184 snRNAs, there were 68 differentially expressed (DE) snoRNAs (64 reduced in OA and four increased in OA), 26 DE snRNAs (24 reduced in OA and two increased in OA), and 90 DE miRNAs (43 reduced in OA and 47 increased in OA) (FDR<0.05 and greater than 10 CPMs in all samples) (Figure 1C; Supplementary Table S3). The most DE miRNAs are shown in Table 1, with snRNAs and snoRNAs shown in Table 2. We further generated a heatmap of the DE miRNAs (Figure 1D) and snRNAs and snoRNAs (Supplementary Figure S4).

**TABLE 1:** Differentially expressed miRNAs with the highest and lowest log2 fold-change when comparing control versus diseased OA anterior cruciate ligament.

miRNAs	Log2 fold-change	False discovery rate adjusted *p*-values
*Upregulated miRNAs in diseased OA ACLs*		
hsa-miR-5100	3.75	6.2E-07
hsa-miR-31-5p	3.14	6.9E-15
hsa-miR-129-5p	2.42	4.0E-03
hsa-miR-144-3p	2.41	3.5E-04
hsa-miR-486-5p	2.33	3.2E-04
hsa-miR-370-3p	2.32	1.4E-06
hsa-miR-543	2.20	6.3E-03
hsa-miR-4521	2.19	5.1E-04
hsa-miR-493-5p	2.17	6.7E-04
hsa-miR-411-3p	2.16	3.9E-03
*Downregulated miRNAs in diseased OA ACLs*		
hsa-miR-206	−6.13	1.9E-06
hsa-miR-12136	−4.35	3.3E-18
hsa-miR-3182	−3.20	3.8E-10
hsa-miR-101-5p	−2.22	9.6E-03
hsa-miR-338-3p	−2.08	1.5E-02
hsa-miR-335-5p	−2.03	7.3E-03
hsa-miR-190b-5p	−1.98	2.5E-03
hsa-miR-29c-3p	−1.89	1.1E-02
hsa-miR-103a-5p	−1.86	3.7E-06
hsa-miR-30b-5p	−1.81	1.7E-02

**TABLE 2:** Small nucleolar RNAs (snoRNAs) and small nuclear RNAs (sncRNAs) identified as being differentially expressed between control and anterior cruciate ligaments derived from osteoarthritic joints.

Name	Family	Action	Target RNA and site-specific modification	Log2 fold-change	False discovery rate adjusted p- values	Up or downregulated
SNORD114	C/D BOX	Site-specific 2′-O-methylation	Unknown	3.60	4.7E-07	OA ACL
SNORD113	C/D BOX	Site-specific 2′-O-methylation	Unknown	2.85	9.8E-05	OA ACL
RNU6	Spliceosome	Complex of snRNA and protein subunits that removes introns from a transcribed pre-mRNA		2.85	4.8E-03	OA ACL
SNORD72	C/D BOX	Site-specific 2′-O-methylation	28s rRNA 28S:U4590	1.83	4.2E-02	OA ACL
RNVU1-19	Spliceosome	Complex of snRNA and protein subunits that removes introns from a transcribed pre-mRNA		1.58	4.8E-02	OA ACL
RNU7-19P	Spliceosome	Complex of snRNA and protein subunits that removes introns from a transcribed pre-mRNA		−7.61	4.0E-07	Control ACL
RNU4-59P	Spliceosome	Complex of snRNA and protein subunits that removes introns from a transcribed pre-mRNA		−4.90	1.4E-33	Control ACL
SNORA36B	H/ACA box	H/ACA family of pseudouridylation guide snoRNAs	18s rRNA 18S:U105 and U1244	−4.25	2.7E-06	Control ACL
SNORA53	H/ACA box	H/ACA family of pseudouridylation guide snoRNAs	Unknown	−3.68	5.1E-15	Control ACL
SNORA73B	H/ACA box	H/ACA family of pseudouridylation guide snoRNAs	Unknown	−3.61	3.9E-05	Control ACL

### 3.3 Pathway analysis of differentially expressed miRNAs

**FIGURE 2:**
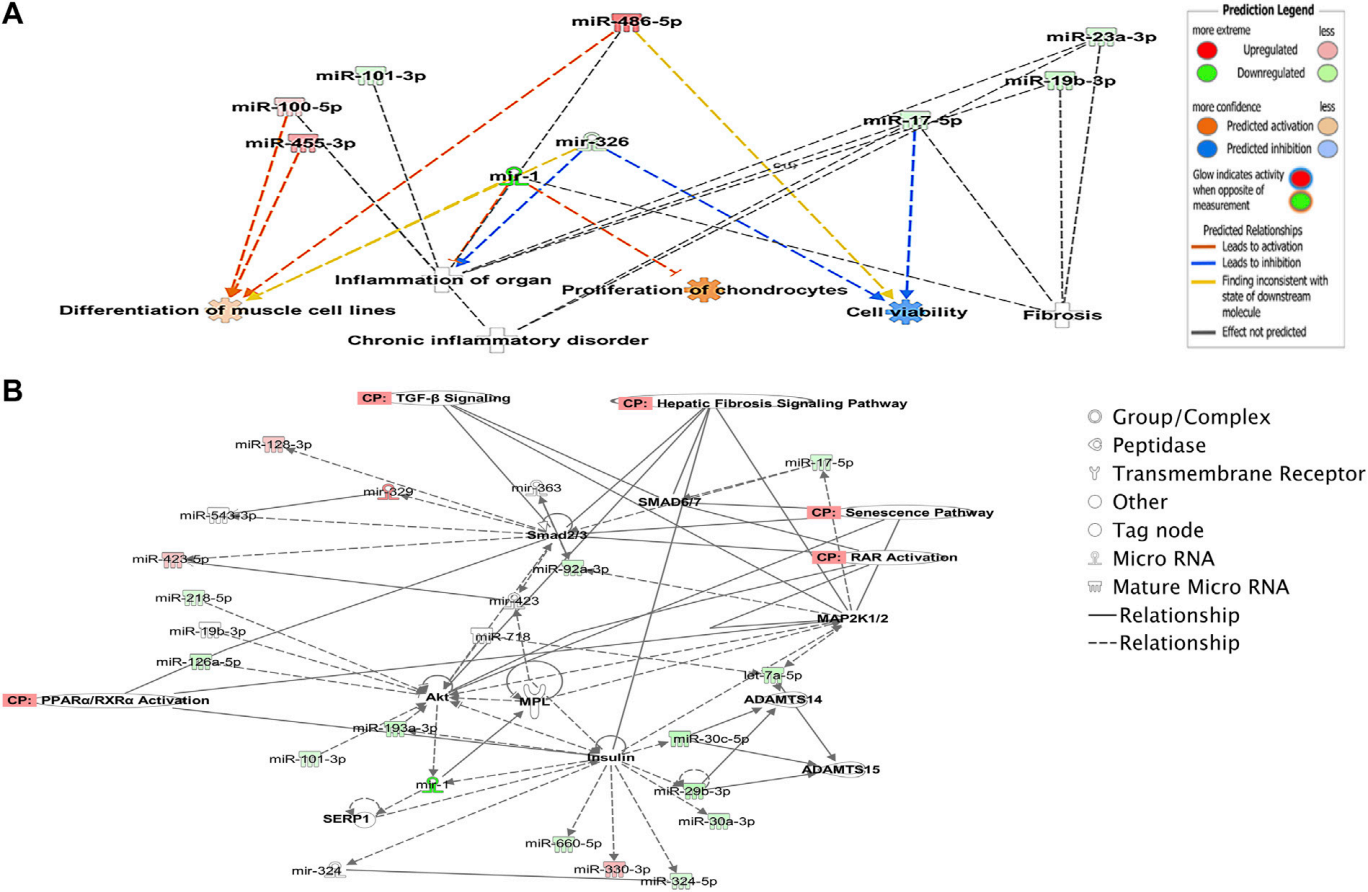
Ingenuity Pathway Analysis-derived functions of differentially expressed miRNAs in diseased OA anterior cruciate ligaments. **(A)** Ingenuity Pathway Analysis identified that cellular functions such as differentiation of muscle, inflammation, proliferation, cell viability, and fibrosis were associated with the differentially expressed miRNAs. Figures generated are graphical representations of molecules identified in our data in their respective networks. Red nodes; upregulated gene expression in the OA anterior cruciate ligament and green nodes; downregulated gene expression in the OA anterior cruciate ligament. Intensity of color is related to higher fold-change. Legends to the main features in the networks are shown. The color denoting the action is dependent on whether it is predicted to be activated or inhibited. **(B)** Top network identified with canonical pathways overlaid for fibrosis, senescence, TGFβ signaling, RAR activation, and PPAR/RXR activation.

To explore potential biological associations between the 90 DE miRNAs in ACLs derived from OA knee joints, we undertook an Ingenuity Pathway Analysis (IPA) “Core Analysis.” Network-eligible molecules were overlaid onto molecular networks based on information from the Ingenuity Pathway Knowledge Database. Networks were then generated based on connectivity. Gene network-inferred features were determined. Significant cellular functions deduced by the DE miRNAs included differentiation of the muscle (*p* < 0.001), inflammation (*p* < 1.42E-10), proliferation of chondrocytes (*p* < 0.03), fibrosis (*p* < 0.001), and cell viability (*p* < 0.03) (Figure 2A). The top scoring network identified was “Organismal Injury and Abnormalities” (score 43) and included OA-related miRNAs such as miR-206, miR-101, let-7f, miR-455, miR-29b, and miR-29c (Figure 2B).

### 3.4 Pathway analysis of the differentially expressed miRNA-predicted target genes

**FIGURE 3:**
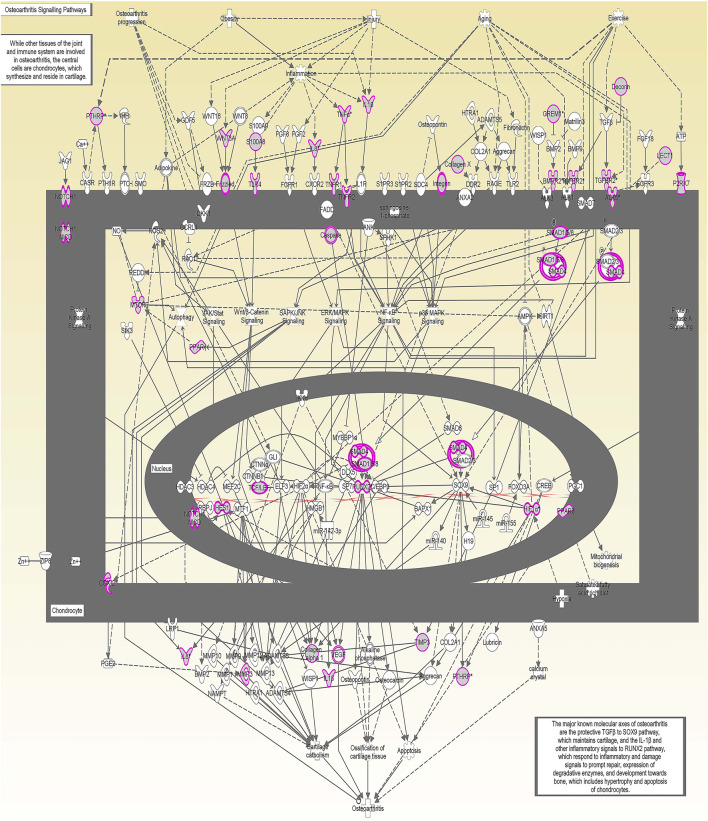
Osteoarthritis (OA) signaling pathway of miRNA-predicted mRNA gene targets. The canonical pathway for OA signaling was highly ranked (*p* = 2.33 E^−23^) using target mRNAs identified in TargetScan from the differentially expressed miRNAs in diseased anterior cruciate ligaments derived from OA patients. The pathway was generated using Ingenuity Pathway Analysis.

**FIGURE 4:**
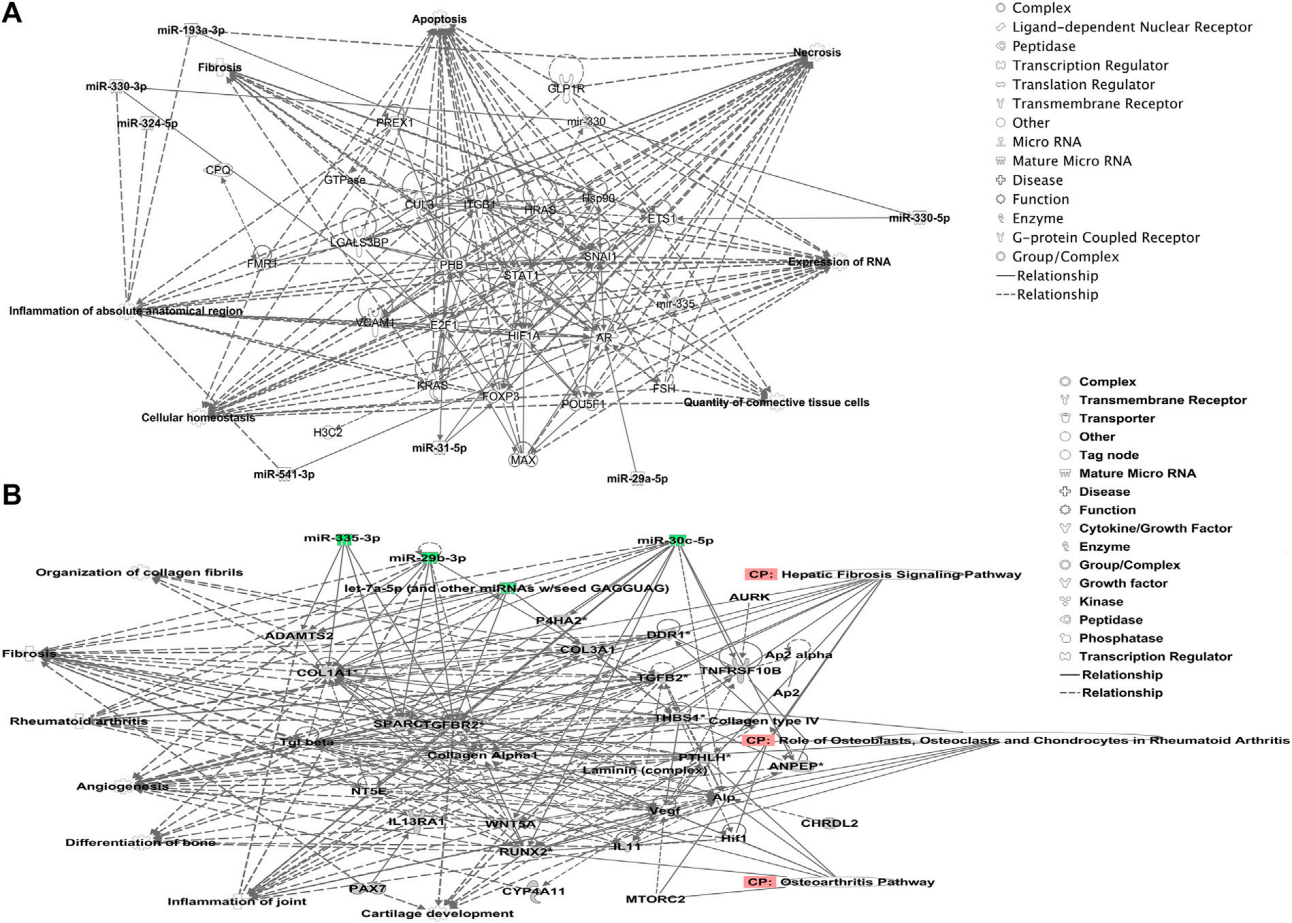
Top-scoring networks derived from the 529 putative mRNAs differentially expressed in anterior cruciate ligaments derived from OA joints. Ingenuity Pathway Analysis (IPA) identified **(A)** “cellular development, movement and genes expression” with a score of 41. **(B)** “Inflammatory disease, organismal injuries and abnormalities” with a score of 35, and within this network are molecules linked to their respective canonical pathways. Both networks **(A, B)** are overlaid with pertinent significant biological functions contained in the gene sets. Figures generated are graphical representations of molecules identified in our data and predicted mRNA targets in their respective networks. Green nodes correspond to downregulated gene expression in anterior cruciate ligaments from OA joints, and red nodes correspond to upregulated gene expression in anterior cruciate ligament from OA joints. Intensity of color is related to a higher fold-change. Legends to the main features in the networks are shown.

**FIGURE 5:**
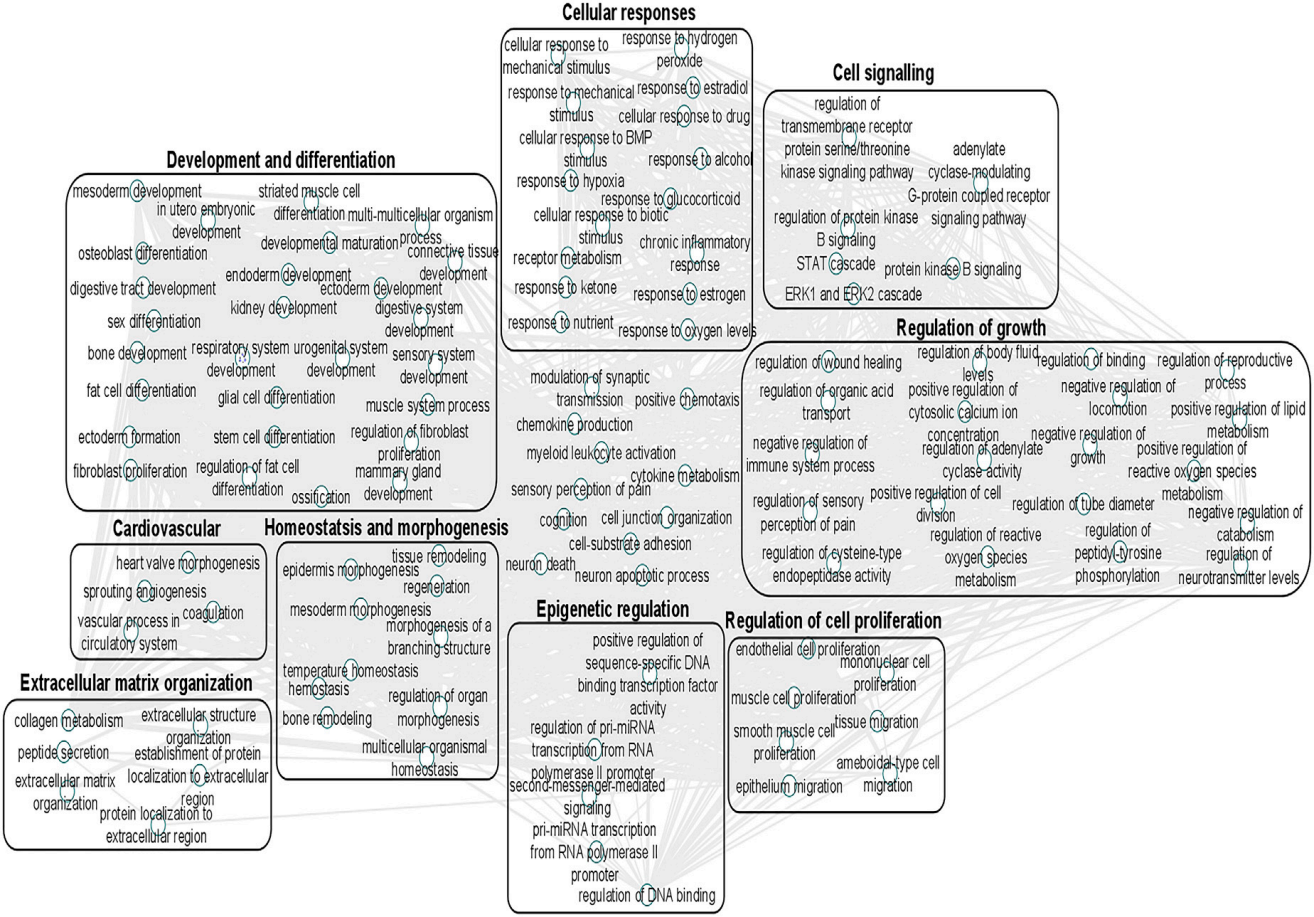
Gene Ontology (GO) biological processes associated with dysregulated miRNA targets were identified following a TargetScan filter module using Ingenuity Pathway Analysis. Gene Ontology terms for biological process (FDR < 0.05) were summarized using ToppGene and visualized using REViGO and Cytoscape. Boxes represent the main clusters of biological processes that were significantly influenced by dysregulated miRNAs between control and diseased OA anterior cruciate ligaments.

We undertook further pathway analysis to determine the mRNA targets of the DE miRNAs. In total, 90 miRNAs that were DE in ACLs derived from OA knee joints compared to controls were initially input into the MicroRNA Target Filter, resulting in 529 mRNAs as putative targets (Supplementary Table S4). These mRNAs were then input into IPA core analysis, and all results are summarized in Supplementary Table S4. The top canonical pathways for target mRNAs of DE miRNAs in OA ACL are provided in Table 3, two of the most significant of which were the osteoarthritis pathway (*p* < 2.3E-23) and hepatic fibrosis (p < 3.1E-32) (Figure 3). The most significant upstream regulators of these mRNAs included tumor necrosis factor (p < 1.3E-101) and transforming growth factor β (TGFβ) (p < 8.5E-83) (Table 4), and the top networks identified are provided in Supplementary Table S4. The network “cellular development, movement and genes expression” (score 41) (Figure 4A) was overlaid with significant biological processes including apoptosis (p < 1.7E-85), fibrosis (p < 1.2E-79), inflammation (p < 3.4E-88), and necrosis (p < 7.2E-88). The network “inflammatory disease” (score 35) (Figure 4B) shows pertinent significant biological processes including organization of collagen fibrils (p < 3.7E-07), fibrosis (p < 2.6E-14), rheumatoid arthritis (p < 3.6E-06), angiogenesis (p < 8.9E-09), differentiation of bone (p < 5E-06), inflammation of the joint (p < 8.8E-07), and cartilage development (p < 1.5E-07). To obtain an overview of pathways of putative target mRNAs, Gene Ontology (GO) tool TOppGene and the biological processes were summarized in REViGO and Cytoscape (Figure 5; Supplementary Table S4).

**TABLE 3:** Top canonical pathways for target mRNAs of differentially expressed microRNAs in diseased osteoarthritic anterior cruciate ligaments.

Name	*P*-value	Overlap (%)
Hepatic fibrosis signaling pathway	1.62E-33	15.8
Hepatic fibrosis/hepatic stellate cell activation	3.06E-32	23.1
Cardiac hypertrophy signaling	1.28E-28	12.3
Colorectal cancer metastasis signaling	1.97E-27	17.4
Role of macrophages, fibroblasts, and endothelial cells	2.32E-27	15.4

**TABLE 4:** Top upstream regulators of differentially expressed microRNAs in diseased osteoarthritic anterior cruciate ligaments.

Name	*p*-value
Tumor necrosis factor	1.31E-101
Hepatic fibrosis/hepatic stellate cell activation	8.50E-83
Transforming growth factor B1	1.14E-81
Lipopolysaccharide	1.45E-77
Tretinoin	9.26E-77

### 3.5 Validation of differential gene expression using qRT-PCR

**FIGURE 6:**
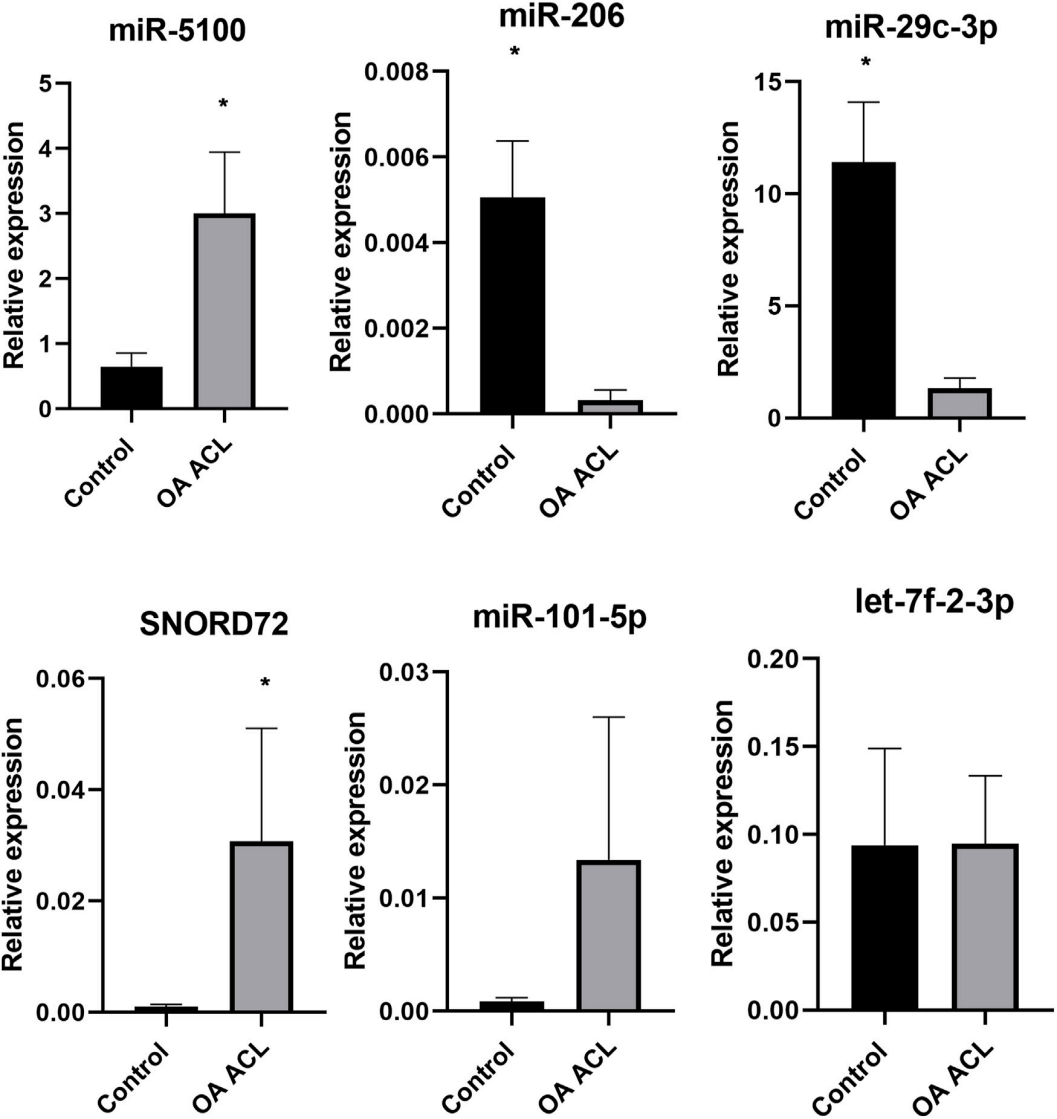
Validation of small RNA sequencing miRNA results using qRT-PCR in an independent cohort. qRT-PCR results show relative gene expression normalized to miR-222, control samples *n* = 4, OA anterior cruciate ligament samples *n* = 4. The Mann–Whitney test was performed using GraphPad Prism v8.0.1, *p < 0.05.

Taking into account the level of differential expression (*p* < 0.05 and logFC > 1.3) and following a literature search, we validated a subset of our DE miRs (Figure 6). Real-time quantitative PCR (qRT-PCR) analyses were undertaken using RNA from an independent cohort (*n* = 6 for control and *n* = 4 for OA ACL samples). In agreement with sequencing data, miR-5100 and SNORD72 were higher expressed in the OA ACL, while miR-206 and miR29c-3p had lower expression in the OA ACL. For two miRs, miR-101 and let-7f, the qRT-PCR findings were not validated.

## 5 Discussion and conclusion

The global prevalence of knee OA is currently 5% and is projected to rise with an increase in the aging population (Hawker, 2019). It is proposed that there is an association between ACL degeneration and subsequent knee OA, suggesting the importance of ACL degradation in the mechanisms of OA pathogenesis (Friel and Chu, 2013). One potential mechanism capable of regulating global alteration in a particular tissue is modification of sncRNA expression (Kaikkonen et al., 2011). To begin to elucidate the role that sncRNAs play in the global changes observed in the ACL during OA and understand further the potential role of the ACL in OA, we undertook a non-biased approach: small RNA sequencing of ACLs from human OA knee joints and compared the findings to those of our control samples derived from human non-OA knee joints. In our previous study, we have identified changing miRNA expression in the aging mice cruciate ligament, which has also been studied in the human osteoarthritic ACL (Li et al., 2017b; Kharaz et al., 2022). However, this is the first time that, to our knowledge, small RNA sequencing has been used in an unbiased manner to interrogate both snoRNAs and miRNAs, which can provide targets for future therapeutic approaches and novel markers for this ACL disease and OA-dependent signatures.

There were 68 snoRNAs, 26 snRNAs, and 90 miRNAs significantly different in ACLs derived from OA knee joints, and the OA status of the donor accounted for the principal variability in the data. Additional bioinformatics analysis was performed to analyze the biological processes and pathways affected by the differentially expressed miRNAs and in addition the putative mRNA targets of the differentially expressed miRNAs, enhancing our understanding of the roles of the dysregulated miRNAs in pathogenesis of diseased OA ACLs.

Several of the DE miRNAs found in this study, including miR-29b, miR -335, miR-424, and miR-941, were previously altered miRNAs in a study comparing ruptured ACLs to diseased OA ACLs (Li et al., 2017a). These miRNAs were found to be correlated with cartilage development and remodeling, extracellular matrix homeostasis, and inflammatory response (Li et al., 2017b). We have found other miRNAs associated with OA including miR-206, miR-5100, and miR-29c, the expression of which is altered in ACLs derived from OA joints and correlated with the validated qPCR results in our current study (Peffers et al., 2018a; Endisha et al., 2018; Malemud, 2018; Zhao et al., 2020).

Pathways identified by the DE miRNAs with known functions in OA in other tissues included inflammation (Lieberthal et al., 2015), proliferation of chondrocytes (Liu et al., 2018), and fibrosis (Remst et al., 2015). Canonical pathways identified have roles in OA pathogenesis including senescence (Jeon et al., 2018), fibrosis (Remst et al., 2015), TGFβ signaling (Davidson Blaney et al., 2007), retinoic acid-binding protein (RAR) activation (Zisakis et al., 2007), and peroxisome proliferator-activated receptor/retinoid X receptor (PPAR/RXR) activation (Li et al., 2017b; Yang et al., 2017). These enriched top signaling pathways and canonical pathways give us further confidence that they may play an important role in the biological processes associated with ACL degeneration and OA development.

To address the roles of miRNAs in diseased OA ACLs, their mRNA target genes were also taken into consideration using IPA and Gene Ontology biological processes, which include extracellular matrix organization, epigenetic regulation, cell signaling, and cell growth and proliferation. In IPA, additional functions affected by these genes, known to have a role in OA pathogenesis and therefore with a potential role in OA ACLs, were highlighted, including apoptosis (Heraud et al., 2000), fibrosis (Remst et al., 2015), inflammation of the joint (Lieberthal et al., 2015), necrosis (Li et al., 2015), organization of collagen fibrils (Hasegawa et al., 2012), angiogenesis (Ashraf and Walsh, 2008), differentiation of bone (Ripmeester et al., 2018), and cartilage development (Goldring and Marcu, 2012). Many canonical pathways enriched by the putative target genes were essential for OA pathogenesis, including the “osteoarthritis pathway.” Additionally, downstream targets of these signaling pathways with known roles in OA pathogenesis were identified and included matrix metalloproteinase-3 (Burrage et al., 2006), tissue inhibitor of metalloproteinase- 3 (Sahebjam et al., 2007), and collagen X α1 (Zhong et al., 2016). Hepatic fibrosis was the most significant canonical pathway identified from the putative mRNAs together with the DE miRNAs in our study. Synovial fibrosis is often found in OA (Remst et al., 2015), and fibrosis has previously been described in OA joints following ACL injury (Douglas et al., 2010). Furthermore, TGFβ, one of the most significant upstream regulators in our mRNA target gene analysis, is the master regulator of fibrosis (Zhong et al., 2016). Many TGFβ-related genes including TGFβ2, TGFβ3, TGFβR1, TGFβR2, and TGFβR3 were predicted targets of the DE miRNAs including miR-98-5p, miR-128-5p, miR-136-3p, and miR-17-5p, strongly implicating it in the fibrosis evident in the diseased ACLs in OA. These findings indicate the potential importance of these pathways in ACL degeneration associated with OA.

Another class of snRNAs, snoRNAs, was altered in the OA ACLs in our study. This conserved class of non-coding RNAs is principally characterized as guiding site-specific post-transcriptional modifications in ribosomal RNA (Dieci et al., 2009). Furthermore, snoRNAs can modify and/or interact with additional classes of RNAs including other snoRNAs, transfer RNAs, and mRNAs (Kishore et al., 2013). A reliable modification site has been assigned to 83% of the canonical snoRNAs, with 76 snoRNAs described as orphan, meaning they act in an unknown or unique manner (Jorjani et al., 2016). Novel functions reported for snoRNAs include modulation of alternative splicing (Khanna and Stamm, 2010), involvement in stress response pathways (Michel et al., 2011), and modulation of mRNA 3′ end processing (Huang et al., 2017). Like miRNAs, snoRNAs are emerging as important regulators of cellular function and OA development (Peffers et al., 2020; Ripmeester et al., 2020; Ali et al., 2021a; Ali et al., 2021b), in part due to their ability to fine-tune the ribosome to accommodate changing requirements for protein production during development, normal function, and disease (Montanaro et al., 2008; van den Akker et al., 2022).

We have previously identified a molecular mechanism for snoRNAs in cartilage aging and OA (Peffers et al., 2020) and their potential use as biomarkers for OA (Steinbusch et al., 2017). Furthermore, other studies have identified that the snoRNAs, SNORD38 and SNORD48, are significantly elevated in the serum of patients developing cartilage damage a year following ACL injury and serum levels of SNORD38 were greatly elevated in patients who develop cartilage damage after ACL injury, suggesting SNORD38 as a serum biomarker for early cartilage damage (Zhang et al., 2012). In addition, we also found an upregulation of SNORD113 and SNORD114 in diseased OA ACLs. These snoRNAs are located in imprinted human loci and may play a role in the evolution and/or mechanism of epigenetic imprinting (Jorjani et al., 2016). They belong to the C/D box class of snoRNAs, and most of the members of the C/D box family direct site-specific 2′-O-methylation of substrate RNAs. However, SNORD113 and SNORD114 differ from C/D box snoRNAs in their tissue-specific expression profiles (including in fibroblasts, osteoblasts, and chondrocytes (Jorjani et al., 2016)) and the lack of complementarity to any RNA. As a result, they are not predicted to guide 2′O-methylation but have novel, unknown roles (Jorjani et al., 2016). Additionally, SNORD113-1 functions as a tumor suppressor in hepatic cell carcinoma by reducing cell growth and inactivating the phosphorylation of ERK1/2 and SMAD2/3 in MAPK/ERK and TGF-β pathways (Xu et al., 2014). We have previously identified that SNORD113-1 expression is also upregulated in OA human knee cartilage but downregulated in aging human knee cartilage, while SNORD114 expression was upregulated in OA knee cartilage (Peffers et al., 2018b).

SNORD72 expression was upregulated in diseased OA ACLs in the small RNA sequencing dataset and validated with an independent cohort using qRT-PCR. In hepatocellular carcinoma, the overexpression of SNORD72 was found to enhance cell proliferation, colony formation, and invasion by stabilizing inhibitor of differentiation (ID) genes which are basic helix–loop–helix (bHLH) transcription factors (Mao et al., 2020). The ID family genes have been shown to play a role in cell proliferation and angiogenesis (Lyden et al., 1999). The lack of a DNA-binding domain results in inhibition of the binding of other transcription factors to DNA in a dominant negative fashion (Benezra et al., 1990). The expression of some members of this family in the rheumatoid arthritis synovium suggests they may have a role in human inflammatory disease (Edhayan et al., 2016). Although the downstream signaling of snoRNAs is principally unknown, snoRNAs regulate ribosome biogenesis (Gong et al., 2017). However a subclass of orphan RNAs does not have complimentary RNA sequences (Sharma et al., 2016). Mao Chet et al., found that ribosome biogenesis was not affected following SNORD72 overexpression, implying it exerts functionality in other ways (Mao et al., 2020). Therefore, while some snoRNAs can regulate the expression of RNAs (Xing et al., 2017), others can reduce the gene stability (Sharma et al., 2016) or directly activate or suppress enzymes (Siprashvili et al., 2016). Together, our findings on snoRNA indicate that changes in ACL snoRNA expression could have important implications in knee OA through both canonical and non-canonical roles.

Our study has a number of limitations. First, while we validated several differentially expressed sncRNAs from our small RNA sequencing data using the qPCR method, we observed a lack of agreement in several other sncRNAs that were also validated but differentially expressed through small RNA sequencing data, which may be due to differences in the unit of measurement between the two methods. The fold change should not be expected to be the same for both methods. This discrepancy may be attributed to the normalization process in data analysis. Future studies will also consider the correlation between qPCR and sequencing results for the genes.

Second, our study was underpowered primarily due to the limited availability of human ACL tissue. Nevertheless, this is the first study of its kind to have used small RNA sequencing to interrogate both snoRNAs and miRNAs in an unbiased manner in healthy and OA diseased human ACLs. Our outcomes demonstrated good statistical power and confirmed the adequacy of the sample size. To ensure that the sample size was representative of the populations being sampled, additional samples were used to validate our results. Future studies in this field would benefit from analyzing larger cohorts of normal and OA diseased human ACL samples, and our study will serve as a platform for sample size estimation for future RNA sequencing of human ACL tissue.

Third, macroscopic grading of knee joint tissues was not performed due to limited images of diseased OA ACL samples; however, images of control samples and according to ICRS scoring (Van den Borne et al., 2007) demonstrated healthy knee joint cartilage with no signs of ACL degeneration.

There was also an imbalance between the sexes in the two groups, with most of the OA-derived ACLs coming from male subjects but all of the control group being sourced from female subjects. We have previously demonstrated that with respect to the human tendon, men and women are transcriptionally different, and gene expression in aged cells moves in opposite directions (Pease et al., 2017). Ligament degeneration has also been demonstrated to be influenced by lower concentrations of sex hormones in young female athletes (Stijak et al., 2015). Finally, there were age discrepancies between the two groups, and so we cannot discount an age effect on sncRNA expression. Age and sex are non-modifiable systematic risk factors for ACL disease and development of OA (Johnson and Hunter, 2014). However, other contributing factors such as underlying disease, obesity, and bone metabolism will also be taken into account in future studies. In addition, the future collection of ACLs from different ages and stages of OA will provide us with a time-related insight into the ligament injury-related small non-coding RNA dysregulation in patients with OA.

In summary, ACL degeneration results in severe physical, social, and economic consequences to the affected individual and leads to development of degenerative joint diseases such as OA. Our study revealed alterations in a number of classes of sncRNAs in ACL tissues derived from patients with knee OA compared to healthy ACLs from non-OA joints. Our functional bioinformatics analyses suggest that the dysregulated miRNAs may regulate cartilage development and remodeling, collagen biosynthesis and degradation, ECM homeostasis, and pathology by interacting with their targets. Uniquely, we also demonstrate that snoRNAs may also have a role in ACL degeneration. Collectively, our study provides novel insights into the ACL-related sncRNA dysregulation in patients with OA, which can be used as potential diagnostic markers and future therapeutic targets to treat ACL degeneration, facilitating prompt positive intervention in the associated development of OA.

## Data Availability

The datasets presented in this study can be found in online repositories. The names of the repository/repositories and accession number(s) can be found in the article/Supplementary Material.
