# Supplementary material for: Small RNA signatures of the anterior cruciate ligament from patients with knee joint osteoarthritis
Source: Front Mol Biosci. 2023 Dec 21;10:1266088. doi: 10.3389/fmolb.2023.1266088 (PMC10768046; doi:10.3389/fmolb.2023.1266088)
Supplement: Supplementary file 4 [file DataSheet1.PDF]

Figure 1. Overview of HiSeq transcriptomics data between control and diseased OA anterior cruciate ligament. A) Categories of RNAs identified in control and diseased OA anterior cruciate ligament B) Principle component analysis revealed that sncRNAs between control and diseased anterior cruciate ligament were distinctly grouped. C) Volcano plot demonstrate significant (FDR< 0.05) differentially expressed sncRNAs (red dots) with a log2 fold change>1.3. D) Heatmap representation of the sncRNA reads from control and OA anterior cruciate ligament. Columns refer to the control and OA anterior cruciate ligament samples and rows of miRNAs identified with their Ensembl identification. The colour of each entry is determined by the number of reads, ranging from yellow (positive values) to red (negative values).

Figure 2. Ingenuity Pathway Analysis derived functions of differentially expressed miRNAs in diseased OA anterior cruciate ligaments. A) Ingenuity Pathway Analysis identified that cellular functions differentiation of muscle, inflammation, proliferation, cell viability and fibrosis were associated with the differentially expressed miRNAs. Figures generated are graphical representations between molecules identified in our data in their respective networks. Red nodes; upregulated gene expression in OA anterior cruciate ligament, and green nodes; downregulated gene expression in OA anterior cruciate ligament. Intensity of colour is related to higher fold-change. Legends to the main features in the networks are shown. The action colour is dependent on whether it is predicted to be activated or inhibited. B) Top network identified with canonical pathways overlaid for fibrosis, senescence, TGFβ signaling, RAR activation and PPAR/RXR activation.

Figure 3. Osteoarthritis (OA) signaling pathway of miRNA predicted mRNA gene targets. The canonical pathway for OA signalling was highly ranked (p=2.33 E^{-23}) using target mRNAs identified in Targetscan from the differentially expressed miRNAs in diseased anterior cruciate ligaments derived from OA patients. The pathway was generated using Ingenuity Pathway Analysis.

Figure 4. Top-scoring networks derived from the 529 putative mRNAs differentially expressed in anterior cruciate ligaments derived from OA joints. Ingenuity Pathway Analysis (IPA) identified: A. 'Cellular development, movement and genes expression' with a scores of 41. (B) 'Inflammatory disease, organismal injuries and abnormalities' with a score of 35 and within this network are molecules linked to their respective canonical pathways. Both networks (A and B) are overlaid with pertinent significant biological functions contained in the gene sets. Figures generated are graphical representations between molecules identified in our data and predicted mRNA targets in their respective networks. Green nodes correspond to downregulated gene expression in anterior cruciate ligaments from OA joints and red nodes correspond to upregulated gene expression in anterior cruciate ligament from OA joints. Intensity of colour is related to higher fold-change. Legends to the main features in the networks are shown.

Figure 5. Gene ontology (GO) biological processes associated with dysregulated miRNA targets were identified following TargetScan filter module using Ingenuity Pathway Analysis. Gene ontology terms for biological process (FDR < 0.05) were summarized with ToppGene and visualised using REViGO and Cytoscape. Boxes represent the main clusters of biological processes that were significantly influenced by dysregulated miRNA between control and diseased OA anterior cruciate ligaments.

Figure 6. Validation of small RNA sequencing miRNA results using qRT-PCR in an independent cohort. qRT-PCR results show relative gene expression normalised to miR-222, control samples n=4, OA anterior cruciate ligament samples n=4. Mann-Whitney test was performed using GraphPad Prism v8.0.1, *$p<0.05$.
