# Supplementary material for: Small RNA signatures of the anterior cruciate ligament from patients with knee joint osteoarthritis
Source: Front Mol Biosci. 2023 Dec 21;10:1266088. doi: 10.3389/fmolb.2023.1266088 (PMC10768046; doi:10.3389/fmolb.2023.1266088)
Supplement: Supplementary file 6 [file DataSheet2.DOCX]

**Supplementary Figures**

**Supplementary Figure 1.** **Donor age and the baseline of small-noncoding RNA expression in control and diseased osteoarthritic (OA) anterior cruciate ligament (ACL) samples.** A) Control age group compared to diseased OA group. B) Principle component analysis (PCA) plot demonstrating the strong significance of OA impact as a major contributor to the expression data's dispersion. Values are mean ± standard deviation. Statistical analysis was undertaken using a Mann Whitney test in GraphPad Prism, *p<0.05


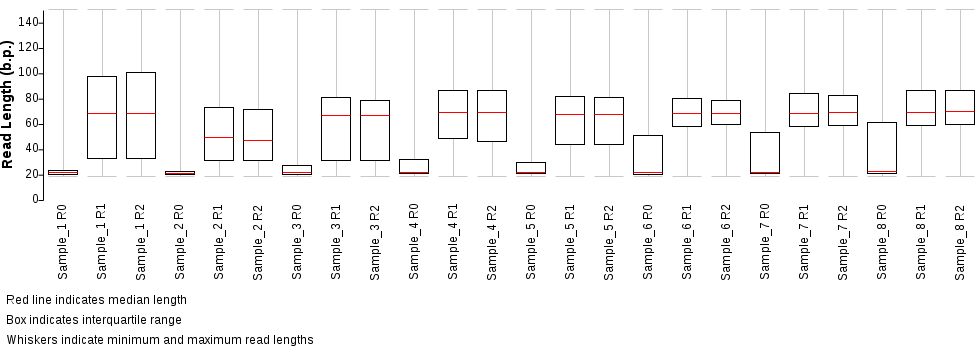


**Supplementary Figure 2**. Read length distribution for all samples after adapter and quality trimming. The box plots depict the distribution of trimmed read lengths for the forward (R1), reverse (R2) and singlet (R0) reads. R0 reads were trimmed more heavily than R1 and R2 reads, suggesting that, after trimming, paired reads are of higher quality than singleton reads.


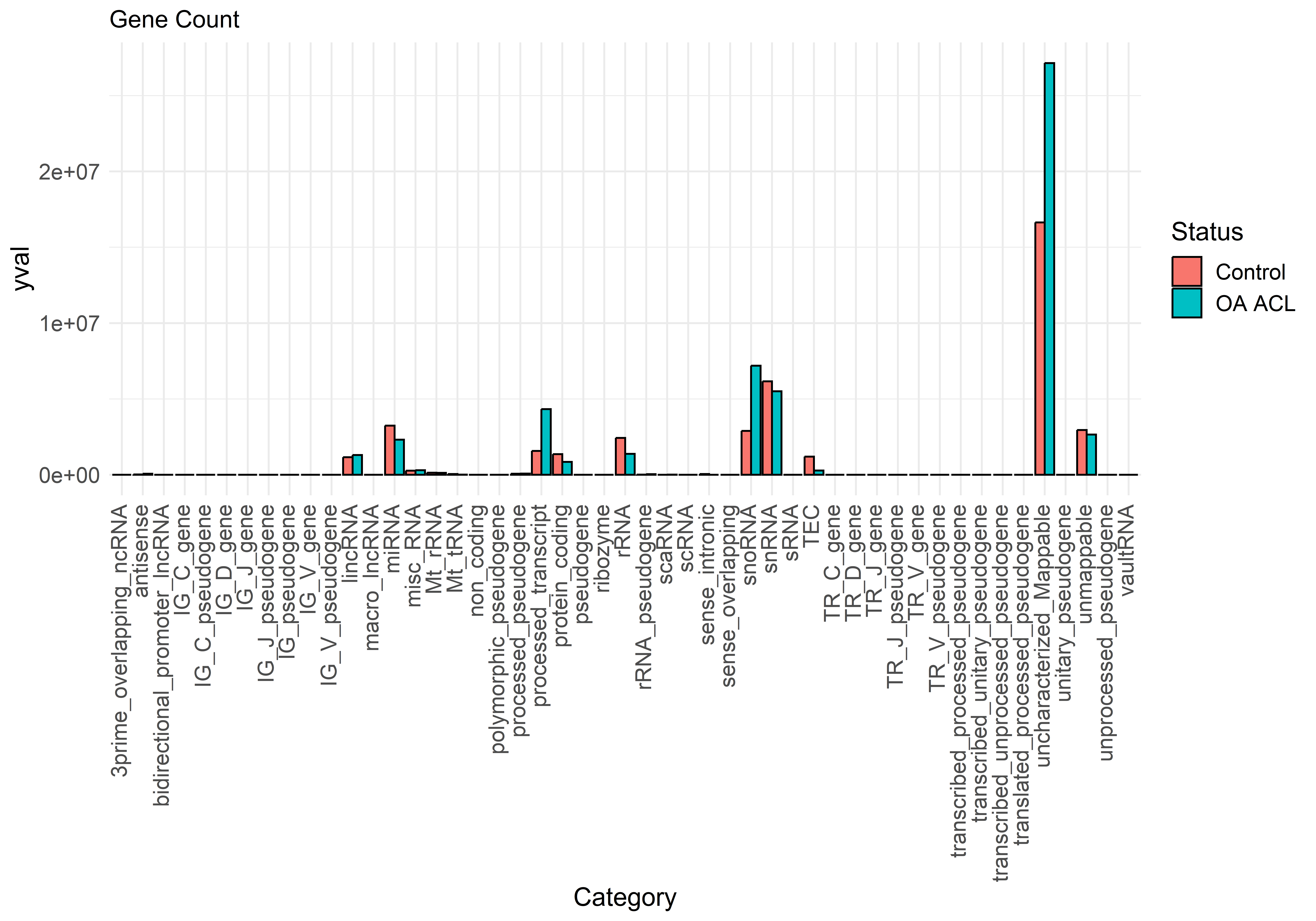
**
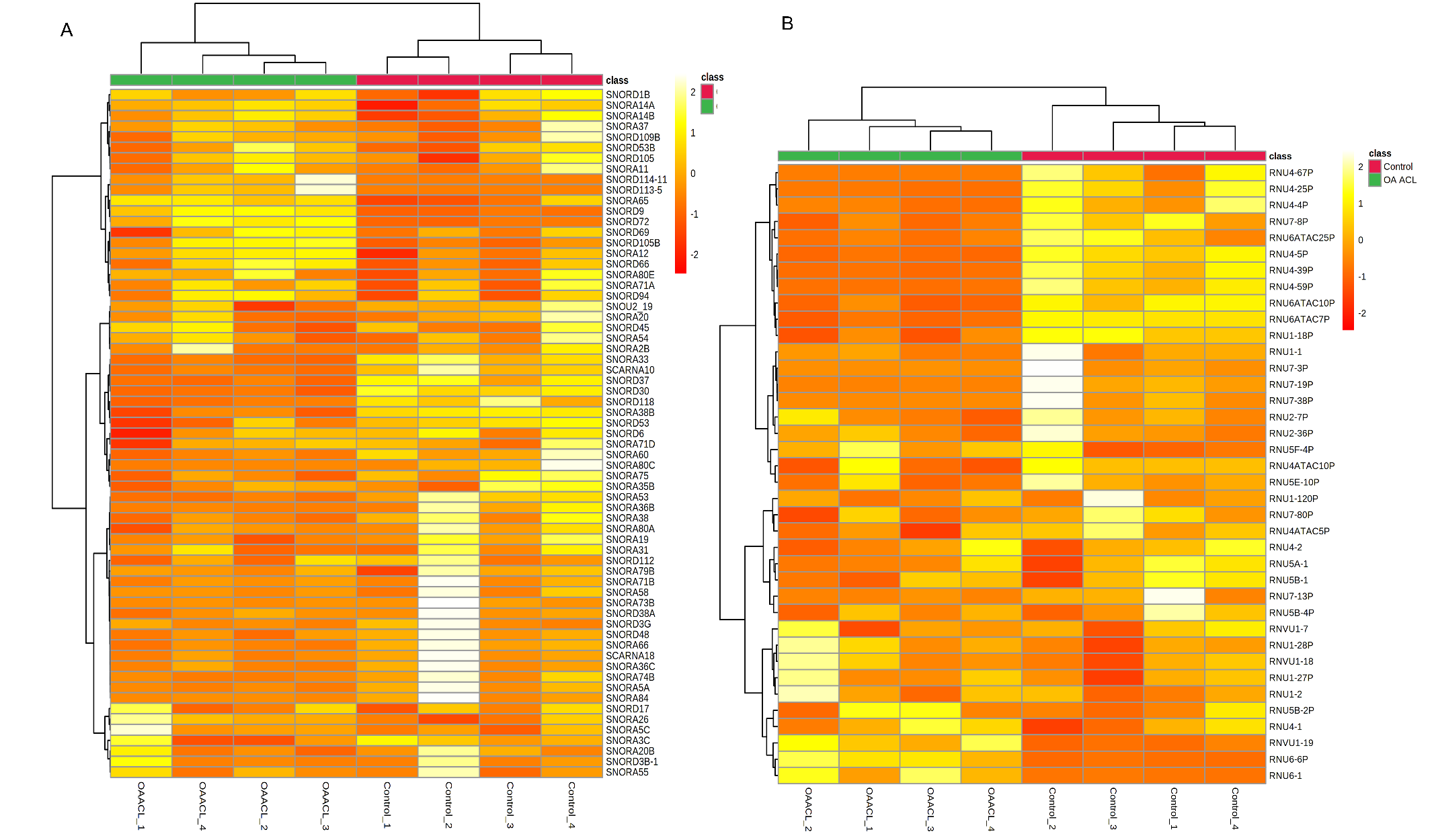
Supplementary Figure 4**. Heatmap representation of the small non-coding RNA reads from control to diseased osteoarthritic (OA) anterior cruciate ligament (ACL) samples. Columns refer to the control and diseased OA ACL samples and rows of (A) small nucleolar RNAs (snoRNAs) and (B) small nuclear RNA (sncRNA) classified by their Ensembl identification. Clustering results shown as a heatmap (distance measure using Euclidean, and clustering algorithm using. The colour of each entry is determined by the number of reads, ranging from yellow (positive values) to red (negative values).

**Supplementary Figure 3.** Distribution of other small RNAs. The proportion/number of reads mapping to each category of RNA is demonstrated for control and OA ACL samples.
